# Social determinants of patiromer adherence and abandonment: An observational, retrospective, real-world claims analysis

**DOI:** 10.1371/journal.pone.0281775

**Published:** 2023-03-27

**Authors:** Nathan Kleinman, Jennifer Kammerer, Kevin LaGuerre, Charuhas V. Thakar

**Affiliations:** 1 Kleinman Analytic Solutions, LLC, Philadelphia, PA, United States of America; 2 Managed Care Health Outcomes, CSL Vifor, Redwood City, CA, United States of America; 3 Medical Information, CSL Vifor, Redwood City, CA, United States of America; 4 Division of Nephrology, University of Cincinnati, Cincinnati, OH, United States of America; The University of Texas MD Anderson Cancer Center, UNITED STATES

## Abstract

**Background:**

Hyperkalemia is a frequent and serious complication in chronic kidney disease (CKD) that can impede continuation of beneficial evidence-based therapies. Recently, novel therapies such as patiromer have been developed to treat chronic hyperkalemia, but their optimal utility hinges on adherence. Social determinants of health (SDOH) are critically important and can impact both medical conditions and treatment prescription adherence. This analysis examines SDOH and their influence on adherence to patiromer or abandonment of prescriptions for hyperkalemia treatment.

**Methods:**

This was an observational, retrospective, real-world claims analysis of adults with patiromer prescriptions and 6- and 12-months pre- and post-index prescription data in Symphony Health’s Dataverse during 2015–2020, and SDOH from census data. Subgroups included patients with heart failure (HF), hyperkalemia-confounding prescriptions, and any CKD stages. Adherence was defined as >80% of proportion of days covered (PDC) for ≥60 days and ≥6 months, and abandonment as a portion of reversed claims. Quasi-Poisson regression modeled the impact of independent variables on PDC. Abandonment models used logistic regression, controlling for similar factors and initial days’ supply. Statistical significance was p<0.05.

**Results:**

48% of patients at 60 days and 25% at 6 months had a patiromer PDC >80%. Higher PDC was associated with older age, males, Medicare/Medicaid coverage, nephrologist prescribed, and those receiving renin-angiotensin-aldosterone system inhibitors. Lower PDC correlated with higher out-of-pocket cost, unemployment, poverty, disability, and any CKD stage with comorbid HF. PDC was better in regions with higher education and income.

**Conclusions:**

SDOH (unemployment, poverty, education, income) and health indicators (disability, comorbid CKD, HF) were associated with low PDC. Prescription abandonment was higher in patients with prescribed higher dose, higher out-of-pocket costs, those with disability, or designated White. Key demographic, social, and other factors play a role in drug adherence when treating life-threatening abnormalities such as hyperkalemia and may influence patient outcomes.

## Background

Although various acute and chronic conditions impact potassium (K^+^) homeostasis, hyperkalemia is a potentially life-threatening electrolyte imbalance often problematic in patients with chronic kidney disease (CKD), heart failure (HF), and diabetes mellitus (DM) [1]. Hyperkalemia can occur in up to 34.6% of patients with CKD and 30% of those with HF [2]. The progressive nature of CKD and HF further increases the risk of hyperkalemia as kidney function declines over time. Treatment with renin-angiotensin-aldosterone system inhibitors (RAASi) has revolutionized the treatment of CKD and HF [1, 3–5]. However, onset of persistent hyperkalemia can impede our ability to offer appropriate evidence-based therapies.

Until recently, sodium polystyrene sulfonate (SPS) was the only treatment for hyperkalemia, which was typically utilized in emergency circumstances or for short-term use. Over the past few years, novel therapies have been developed and approved for chronic management of hyperkalemia. Patiromer, the first novel approved therapy, is a sodium-free, nonabsorbed, daily-use K^+^-binding polymer indicated for treating hyperkalemia. Its use has been supported by the pivotal study OPAL-HK and other efficacy data in diverse, medically complex patients [6, 7]. Clinical and real-world evidence suggests patients are more likely to be on RAASi therapy or at optimal RAASi doses while taking a concurrent K^+^ binder such as patiromer [6–9].

Successful treatment of chronic comorbid conditions, such as CKD or HF, and their complications hinges on medication adherence. Several factors influence patient adherence, including social determinants of health (SDOH). SDOH serve as nonclinical markers of general security, including social support, community connectedness, economic stability, and living environment. The World Health Organization defines SDOH as avoidable inequities in health that arise from circumstances in which people grow, live, work, and age [10], further shaped by political, social, and economic forces. Such factors (eg, income, education, household size/support, environmental safety, proximity to care) may influence health behaviors and correlate with health risks such as medication nonadherence, delays in seeking care, lack of a primary care provider, and toxic stress [10, 11].

Optimal care delivery in chronic conditions such as CKD, HF, or DM is intricately linked with medical therapy, and SDOH. When combined with claims and clinical data, SDOH may enhance complete care planning, address patient-specific barriers by individualizing interventions, and assess the most at-risk groups. The objective of this study is to assess the effects of real-world experience of SDOH on patiromer adherence or abandonment of initial prescription. We aim to achieve our objective by utilizing the strengths of a large national claims-based database, which allows correlation of pharmacy, clinical, and key SDOH parameters. This study was executed in a multi-payer data set that represents a broader real-world population than a single-payer data set. It also incorporated relevant methodologies that reasonably excluded patients who may—in a single-payer data set—switch insurance and be otherwise ineligible for study inclusion. Adherence methodology (PDC) was specifically chosen as the well-accepted approach to HEDIS quality measurement of adherence, whereas prior patiromer studies used “continuous exposure” or modified PDC methodology. In addition, this is the first known assessment of abandonment patterns, which indicate a different barrier to access where a patient has successfully been prescribed patiromer but never started it, for unknown reasons.

## Material and methods

### Materials and study design

This was an observational, retrospective analysis of data on adults with prescription claims, using a predefined data extract from Symphony Health Analytics claims. Census data supplemented ZIP Code–based regional information at a population level, providing insights on population density, race, per-capita income, education, poverty level, and disability status.

This retrospective, administrative claims database analysis was based on historic deidentified patient data and did not involve patients directly; therefore, institutional review board/ethics committee approval was not necessary or applicable.

### Patient population

The adherence analysis included patients with a final approved patiromer claim during 2015–2018, and the abandonment analysis included those with a final approved or reversed patiromer claim during 2015–2018. Patients were at least 18 years old 6 months prior to the index date, which was defined as the first patiromer fill date, and all patients had at least 6 months of pre-index and 12 months of post-index (any) prescription activity.

Subgroups evaluated included those with HF, hyperkalemia-confounding medications, and distinct stages of CKD (International Classification of Diseases, 10th edition–based CKD stages 1–2 vs CKD stages 3–4 vs CKD stage 5/end-stage renal disease [ESRD]).

### Outcomes and variables

The primary adherence outcome was proportion of days covered (PDC). To identify SDOH associations with starting or continuing patiromer, patients were considered adherent if the PDC calculated over 60 days was >80%. To broadly gauge and describe chronicity of patiromer use once started, PDC was also calculated over 6 months.

Abandonment was defined as the proportion of reversed initial patiromer claims among all reversed or approved claims with no evidence of a subsequent additional adjudicated paid claim. This was applied to only a first patiromer fill without prior history of patiromer in the look back period and did not include any fill attempts after the first (initial) fill. Thus, we defined an indirect measure of abandonment by representing the frequency of prescriptions never picked up once filled.

The following population-descriptive statistics were identified for study patients: gender; age; region (based on the first digit of a patient’s ZIP Code); initial prescription plan type; initial patiromer dosage strength; initial prescription mode of transmission; initial prescriber specialty; pre-index number of daily-use prescription medications, use of other K^+^ binders, or hyperkalemia diagnosis; and post-index patiromer out-of-pocket cost per day supplied, non-patiromer out-of-pocket cost/month. The patient 3-digit ZIP Code was linked to census-based averages for employment status, race, poverty level, disability, education, per-capita income, and population density.

### Statistical methods

Both 60-day and 6-month PDC values were characterized using quasi-Poisson regression models. Quasi-Poisson models were chosen because they account for overdispersion in the days covered count data. Independent variables were controlled for in the regression models to assess and measure their impacts on PDC. These variables included the patient, prescription, provider, plan, and regional census factors listed above, as well as post-index K^+^-confounding medications and post-index medical claim activity for HF, CKD, and ESRD. Prescription K^+^-confounding medications were grouped by class and included RAASi, K^+^ supplements, nonsteroidal anti-inflammatory drugs, immunosuppressants, K^+^-increasing antibiotics, and others (amiloride, sacubitril/valsartan, and triamterene). Only one regional census variable could be included in the regression model at a time due to collinearity issues.

Prescription abandonment was modeled using logistic regression, controlling for factors similar to those in the adherence models in addition to initial dispensed days’ supply and pharmacy type.

The regression models were run using RStudio version 1.2.5042 [12]. Statistical significance in all regression models was set at p-value <0.05.

This study used observational analysis of an aggregate dataset where the data are anonymized and considered exempt from further informed consent.

## Results

**Fig 1:**
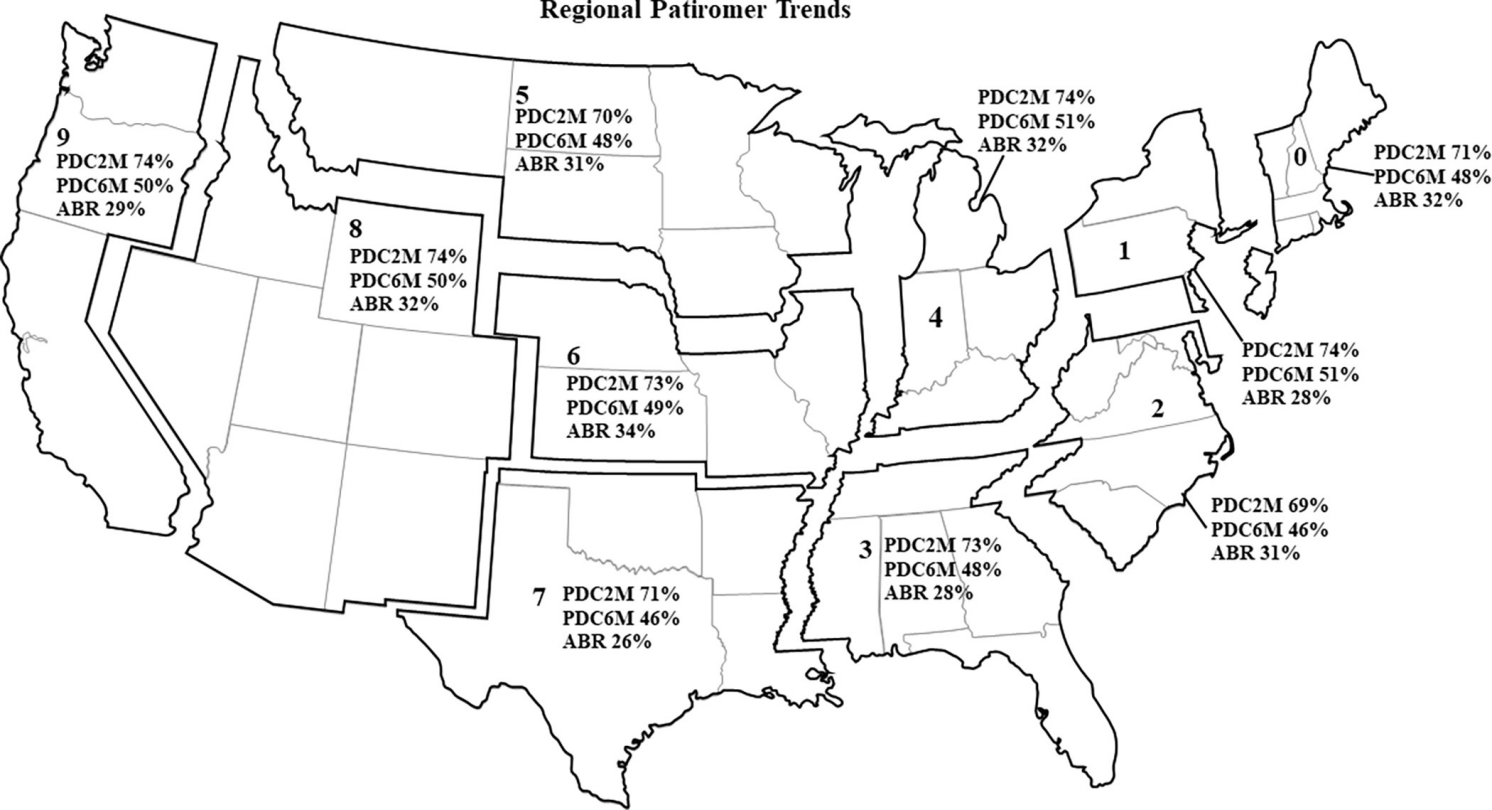
Patiromer prescribing, adherence, and abandonment rates by region. n = patiromer patients (% total). Numbers 0–9 indicate the first digit of a region’s ZIP code. Abbreviations: ABR, abandonment rate; PDC2M, 60-day proportion of days covered; PDC6M, 6-month proportion of days covered. Adapted from iStock image. https://www.istockphoto.com/vector/united-states-of-america-map-us-blank-map-template-outline-usa-map-background-gm1301588831-393587962.

In the adherence analysis, initially 33,329 patients had an approved patiromer prescription claim, of which 33,250 met age requirements and 24,405 met all requirements (index, age, claim activity). Average age was 63±13 years, and 41% of the population was female. Patients were well represented across the United States, with the heaviest prescribing in the southeastern, southern, and western areas (Fig 1).

In the pre-index period, defined as that before starting patiromer, patients averaged 9.9±5.4 unique daily-use medications and $47.19±$96.30 in out-of-pocket costs per month on all daily-use prescription medications. Twenty-five percent of patients had a pre-index SPS prescription, with an average gap of 53±49 (median 35) days between last SPS fill and first patiromer fill (Table 1).

**Table 1:** Patiromer adherence population descriptive outcomes.

Patiromer Patients (N = 24,405)	Mean±SD or Count (%)
**6-Month Pre-Index**	
Number of unique daily-use medications	9.9±5.4
Average total out-of-pocket per month, any (non-patiromer) prescription, USD	47.19±96.30
Patients with SPS use	5,991 (25)
Average days from last SPS prescription fill date to patiromer start	52.9±48.9 (median 35 days)
**6-Month Post-Index**	
Average number of fills per patient	2.9±2.0
Average days’ supply per prescription	34.4±17.3
Patients with initial patiromer dose of 8.4 g	22,453 (92)
Average number of dose changes	0.070±0.31
Average out-of-pocket per patiromer prescription, USD	52.48±166.84
Average patiromer out-of-pocket cost per day supplied, USD	1.79±5.22
Average total out-of-pocket per month, any non-patiromer prescription, USD	46.34±152.86
Number of unique daily-use medications	10.8±5.4
Average 60-day patiromer PDC, %	72.9±0.26
PDC >80%	11,812 (48)
PDC >90%	10,490 (43)
Average 6-month patiromer PDC, %	49.4±0.31
PDC >80%	6,180 (25)
PDC >90%	4,060 (17)

Abbreviations: PDC, proportion of days covered; SD, standard deviation; SPS, sodium polystyrene sulfonate; USD, United States dollars.

In the post-index analyses (defined as after initiating patiromer), most (92%) patients started patiromer at a dose of 8.4 g (with few [0.07±0.30] dose changes). Average number of fills was 2.9±2.0, while average days’ supply was 34.4±17.3 days. Patients showed an increase of about one prescription in mean number of unique daily-use medications from pre-index (9.9±5.4) to post-index (10.8±5.4). Mean out-of-pocket cost for a patiromer prescription was $52.48±$166.84, averaging out to $1.79±$5.22 per day supplied (Table 1), while average out-of-pocket spending on non-patiromer prescriptions remained similar at $46.34±$152.86.

Average 60-day PDC was 72.9%; 48% of the patients had 60-day PDC >80%. At 6 months, average PDC was lower (49.4%), and 25% were adherent with PDC >80% (Table 1). However, adherence did not appear to be dose-dependent.

A number of independent variables in the adherence regression models had a significant association with PDC, including the region of the country in which the patient lived. At 60 days, adjusted patiromer PDC remained relatively high throughout all regions (range: 69% in region 2 to 74% in regions 1, 4, 8, and 9; Fig 1). At 6 months, adjusted PDC ranged from 46% to 51% across regions (Fig 1).

**Fig 2:**
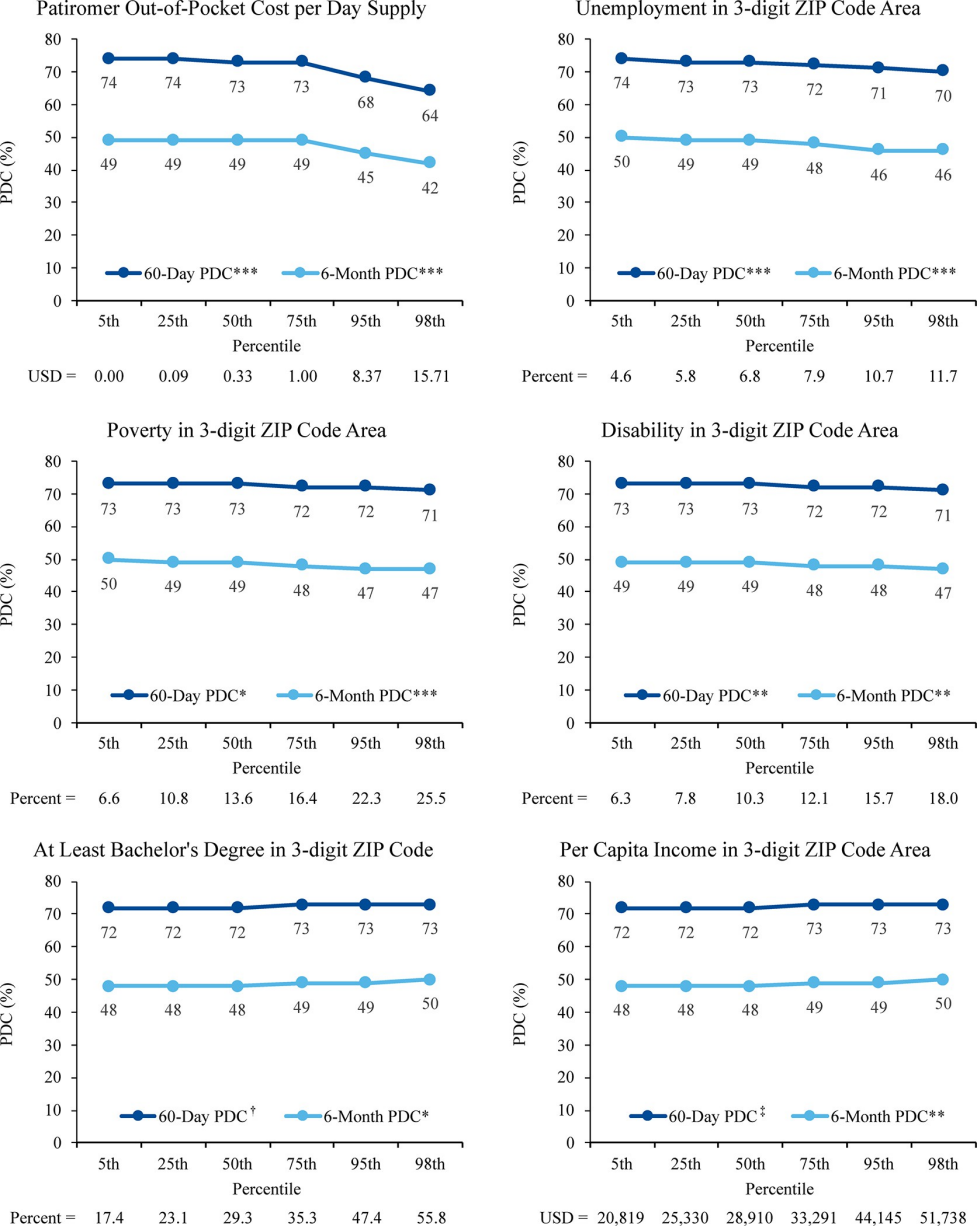
60-day and 6-month regression-adjusted PDC for continuous independent variables. ***p<0.001; **p<0.01; *p<0.05; ^†^p = 0.11; ^‡^p = 0.17. Abbreviations: PDC, proportion of days covered; USD, United States dollars.

Adjusted mean PDC values for additional significant categorical variables are shown in Table 2. Holding other variables in the model constant, those variables favoring high PDC included older age, male gender, Medicare coverage, initial prescription by nephrologist (vs generalist or cardiologist), and recent hyperkalemia diagnosis or SPS treatment (Tables 2 and 3). For continuous independent factors associated with PDC, Fig 2 illustrates association of adherence with regionally higher per capita income and concentrations of advanced education (bachelor’s or master’s degree).

**Table 2:** 60-day and 6-month regression-adjusted proportion of days covered (N = 24,405).

	N	Adjusted 60-Day PDC, %	p-Value	Adjusted 6-Month PDC, %	p-Value
**Demographic variables**					
Age <45 (ref)	2,312	69		43	
45 ≤ Age <55	3,451	71	<0.001	47	<0.001
55 ≤ Age <65	6,262	72	<0.001	49	<0.001
65 ≤ Age <75	6,964	74	<0.001	50	<0.001
Age ≥75	5,416	74	<0.001	50	<0.001
Female	9,885	71	<0.001	47	<0.001
Male (ref)	14,520	73		50	
ZIP Code					
1st digit = 0	1,529	71	<0.001	48	0.007
1st digit = 1	2,780	74	0.83	51	0.42
1st digit = 2	2,114	69	<0.001	46	<0.001
1st digit = 3	4,404	73	0.03	48	<0.001
1st digit = 4	1,903	74	0.70	51	0.46
1st digit = 5	517	70	0.004	48	0.11
1st digit = 6	1,678	73	0.15	49	0.31
1st digit = 7	4,242	71	<0.001	46	<0.001
1st digit = 8	1,448	74	0.98	50	0.69
1st digit = 9 (ref)	3,777	74		50	
**Initial patiromer prescription strength**					
8.4 g (ref)	22,453	72		48	
16.8 g	1,798	74	0.002	49	0.20
25.2 g	154	71	0.42	49	0.81
**Initial patiromer prescription transmission mode**					
Electronic (ref)	1,432	68		43	
Paper	1,506	70	0.07	46	0.06
Oral/IVR	7,102	69	0.68	45	0.27
Fax	1,194	73	<0.001	48	<0.001
New from existing prescription	295	66	0.03	43	0.21
Other transmission	12,876	75	<0.001	52	<0.001
**Pharmacy plan type**					
PBM	2,696	71	<0.001	47	<0.001
Cash	611	64	<0.001	38	<0.001
Employer/Commercial	2,805	69	<0.001	44	<0.001
Medicaid	1,972	72	<0.001	49	0.05
Medicare (ref)	13,986	75		51	
Other	2,335	70	<0.001	45	<0.001
**Initial prescriber classification and specialization**					
Internal medicine; nephrology (ref)	17,054	74		50	
Generalists	3,751	69	<0.001	45	<0.001
Internal medicine; cardiovascular disease	404	72	0.07	48	0.12
Other	3,196	69	<0.001	45	<0.001

(ref) indicates categorical reference group.

Abbreviations: IVR, interactive voice response; PBM, pharmacy benefits manager; PDC, proportion of days covered.

**Table 3:** Concurrent confounding conditions in adherence study population.

	Count (N)	Adjusted 60-Day PDC, (%)	p-Value	Adjusted 6-Month PDC (%)	p-Value
**6-Month Pre-Index Factors**					
SPS Rx	5,991	74	<0.001	51	<0.001
No SPS Rx (ref)	18,414	72		48	
Hyperkalemia Dx	6,924	74	<0.001	51	<0.001
No Hyperkalemia Dx (ref)	17,481	72		48	
**Specific 6-Month Post-Index Conditions**					
No HF (ref)	21,055	73		49	
HF (no CKD/ESRD)	477	74	0.73	49	0.08
HF and CKD 1–2	1,200	73	0.65	46	0.03
HF and CKD 3–4	1,335	72	0.01	47	<0.001
HF and (CKD 5 or ESRD)	1,538	70	<0.001	43	<0.001
**6-Month Post-Index Confounding Medication Use**					
RAASi Rx	11,033	73	0.006	49	<0.001
No RAASi Rx (ref)	13,372	72		48	
Potassium Supplement Rx	372	68	0.002	37	<0.001
No Potassium Supplement Rx (ref)	24,033	73		49	
NSAID <28-day Rx	1,079	73	0.95	49	0.30
No <28-day NSAID Rx (ref)	23,326	72		48	
NSAID ≥28-day Rx	1,213	71	0.06	48	0.36
No ≥28-day NSAID Rx (ref)	23,192	73		49	

(ref) indicates categorical reference group.

Abbreviations: CKD, chronic kidney disease; Dx, diagnosis; ESRD, end-stage renal disease; HF, heart failure; NSAID, nonsteroidal anti-inflammatory drug; PDC, proportion of days covered; RAASi, renin-angiotensin aldosterone system inhibitor; Rx, prescription; SPS, sodium polystyrene sulfonate.

In the multivariate analysis, the lowest PDC rates were observed in patients in eastern and southern regions (ZIP 0, 2, and 7) and were associated with cash-paid transactions and those with comorbid HF and advanced stages of renal dysfunction (Tables 2 and 3). Patiromer adherence declined where unemployment, poverty, and disability were higher, but adherence was most adversely affected by patiromer out-of-pocket costs per days supplied (Fig 2).

**Fig 3:**
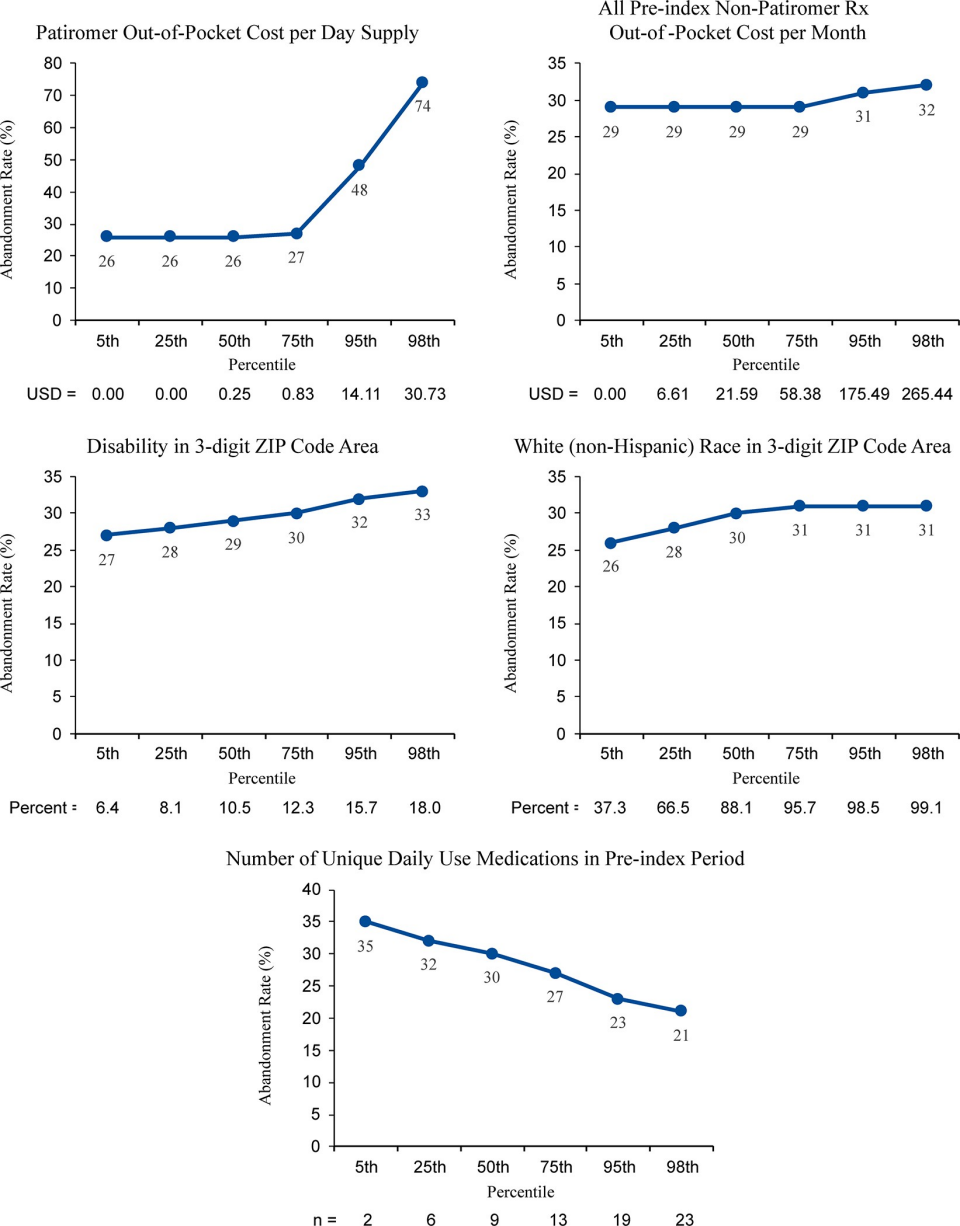
Regression-adjusted abandonment rate for first patiromer prescriptions (all p<0.001). Abbreviations: USD, United States dollars.

Only half of patients on patiromer were also on RAASi (Table 3). Patiromer-specific out-of-pocket costs significantly influenced adherence (PDC), in contrast to either total (non-patiromer) medication costs or pre-index total number of daily use medications (Fig 3).

In the abandonment analysis, 36,185 patients had an approved or reversed patiromer claim, of which 36,110 met age criteria and 25,762 met all inclusion criteria. The average age was 63±13 years, and 41% were female. Regional distribution was similar to that shown in Fig 1.

Table 4 provides regression-adjusted abandonment rates for categorical variables. Abandonment ranged from 26.0% to 34% throughout regions. Holding other variables constant, groups with the lowest abandonment (i.e., the groups most likely to pick up a prescription once filled) were those who were age 55 to <65 years, were in region 7 (south central), were covered by Medicaid or commercial/employer plans, or had a recent hyperkalemia diagnosis (Tables 3 and 4). Unexpectedly, abandonment was less likely in patients with a greater baseline medication burden (Fig 3).

**Table 4:** Regression-adjusted percent of first patiromer prescriptions abandoned (N = 25,762).

	N	Regression-Adjusted Abandonment Rate (%)	p-Value
**Demographic variables**			
Age <45 years (ref)	2,430	31	
45 ≤ Age <55 years	3,499	29	0.11
55 ≤ Age <65 years	6,259	28	0.003
65 ≤ Age <75 years	7,806	31	0.78
Age ≥75 years	5,768	29	0.18
Female	10,473	29	0.06
Male (ref)	15,289	30	
ZIP Code			
1st digit = 0	1,335	32	0.09
1st digit = 1	2,633	28	0.45
1st digit = 2	2,184	31	0.07
1st digit = 3	4,802	28	0.64
1st digit = 4	2,099	32	0.04
1st digit = 5	659	31	0.38
1st digit = 6	2,061	34	<0.001
1st digit = 7	4,679	26	0.005
1st digit = 8	1,618	32	0.02
1st digit = 9 (ref)	3,692	29	
**Days’ supply**			
Supply <28 days	1,537	29.9	0.60
Supply ≥28 days and ≤30 days (ref)	20,914	29.3	
Supply >30 days	3,311	29.8	0.54
**Pharmacy type**			
Retail (ref)	17,082	31	
Mail/specialty	4,536	32	0.15
Non-retail and non-mail/specialty	4,144	23	<0.001
**Pharmacy plan type**			
PBM	1,986	30	0.104
Cash	768	28	0.03
Employer/commercial	2,713	22	<0.001
Medicaid	1,596	23	<0.001
Medicare (ref)	16,322	32	
Other	2,377	25	<0.001
**Prescription strength**			
8.4 g (ref)	23,773	29	
16.8 g	1,826	34	<0.001
25.2 g	163	36	0.05
**Initial prescriber classification and specialization**			
Internal medicine; nephrology (ref)	18,000	29	
Generalists	3,969	29	0.47
Internal medicine; cardiovascular disease	415	32	0.29
Other	3,378	30	0.72

(ref) indicates categorical reference group.

Abbreviations: PBM, pharmacy benefits manager.

Factors associated with the highest rates of abandonment (groups least likely to pick up a prescription once filled) resided in region 6 (Midwest), had Medicare coverage, and had starting doses above 8.4 g (Table 4). Abandonment was also higher where either patiromer or non-patiromer out-of-pocket costs were higher and in regions with greater concentrations of disabled or White populations (Fig 3). Gender, comorbidities, and concomitant hyperkalemia-confounding medications had little or no influence on abandonment rates (Tables 4 and 5).

**Table 5:** Concurrent confounding conditions in the abandonment analysis study population.

	N	%	p-Value
**Specific 6-Month Pre-Index Factors**			
SPS Rx	6,103	30	0.42
No SPS Rx (ref)	19,659	29	
Hyperkalemia Dx	7,232	28	0.001
No Hyperkalemia Dx (ref)	18,530	30	
No HF (ref)	22,387	29	
HF (no CKD/ESRD)	525	31	0.39
HF and CKD 1–4	1,557	30	0.37
HF and (CKD 5 or ESRD)	1,293	31	0.32
**6-Month Pre-Index Confounding Medication Use**			
RAASi Rx	13,692	30	0.42
No RAASi Rx (ref)	12,070	29	
Potassium Supplement Rx	554	30	0.64
No Potassium Supplement Rx (ref)	25,208	29	
NSAID <28-day Rx	1,088	28	0.31
No <28-day NSAID Rx (ref)	24,674	29	
NSAID ≥28-day Rx	1,432	28	0.33
No ≥28-day NSAID Rx (ref)	24,330	29	

(ref) indicates categorical reference group

Abbreviations: CKD, chronic kidney disease; Dx, diagnosis; ESRD, end-stage renal disease; HF, heart failure; NSAID, nonsteroidal anti-inflammatory drug; RAASi, renin-angiotensin aldosterone system inhibitor; Rx, prescription; SPS, sodium polystyrene sulfonate.

## Discussion

Since the release of novel daily-use agents to treat hyperkalemia, this is among the first real-world analyses describing abandonment of or adherence to K^+^-binder prescriptions. Moreover, the analysis utilizes the strengths of claims data to examine the effect of SDOH on these two critical outcomes to inform opportunities that may improve outcomes in often-complex comorbid conditions.

Our analysis examined the impact of SDOH, demographic factors, and comorbidities by looking at K^+^-binder use in two ways: i) adherence based on PDC; and ii) abandonment or proportion filled at a pharmacy but never picked up by the patient. First, while the mean 60-day PDC was 72.9%, 6-month PDC dropped to 49.4%; both rates were below the Centers for Medicare and Medicaid Services–defined threshold for long-term adherence with chronic medications [13]. The portion of patients achieving the PDC >80% threshold at 60 days and 6 months was 48.4% and 25.3%, respectively. Although patiromer was studied for chronic daily use, long-term use in practice appears limited, suggesting an important opportunity to identify and address the barriers to not only continuing treatment once started but also those preventing patients with a prescription from starting it. In multivariate analyses, the lowest PDC rates were observed in patients in the eastern and southern regions; associated with cash-paid transactions; and in those with HF particularly with comorbid advanced CKD stages. Patiromer adherence declined where unemployment, poverty, and disability were higher, but was most adversely affected by patiromer out-of-pocket costs per days supplied. Interestingly, neither medication burden prior to use of patiromer nor out-of-pocket costs for other prescriptions influenced PDC.

Another observation was that PDC was higher when patiromer was initially prescribed by a specialist, which may reflect the relationship or communication quality. Beyond nonadherence, some medication-specific flags of low literacy that may be tackled include frequently missed appointments, identifying medications by sight rather than label, inability to name a medication, how to use it or its purpose, and asking few questions [14]. For patiromer patients, providers may consider demonstrating how to mix and use it, describing need-to-know points in plain language, and using teach-back methods to ensure understanding [14]. This combined with the findings above regarding 6-month PDC indicate that different patient and physician education or awareness strategies are needed to improve long-term adherence in the treatment of chronic conditions.

Abandonment rates, defined as proportion of prescriptions never picked up once filled, ranged between 22% to 34% depending on baseline patient characteristics. Patients with the lowest rates of abandoning a patiromer prescription were those who were prescribed the lowest commercially available dose of patiromer (8.4 g) in age groups 55 to <65, living in region 7 (South Central), had drug coverage under Medicaid or commercial/employer plans, or had a recent hyperkalemia diagnosis. Abandonment was greater with higher out-of-pocket costs or in geographic areas where a higher proportion of the population reported disability. Daily medication complexity, prescription cost, and age-related cognitive changes are often thought to be substantial pressures affecting medication adherence [15]. In this study, patiromer adherence was lowest in the youngest age group and highest in the 65 years and older group, consistent with (but perhaps counterintuitive) other data showing higher adherence in older patients [15]. Regions that had patients with more upper education and higher income were more likely to abandon an original patiromer prescription, but also showed greater adherence rates after starting. We speculate that higher health literacy may favor self-management or ability to achieve lifestyle or dietary changes to counter hyperkalemia before or instead of drug treatment [16].

Our observation suggests that patiromer was more heavily prescribed in regions that also included the OPAL-HK [7] pivotal trial study sites (CA, FL, GA, MO, NY, PA, TX) and, like the OPAL-HK study population, patients were more likely to be older, Medicare-eligible men with substantial polypharmacy indicative of multiple chronic conditions. Out-of-pocket spending for prescription drugs appears to be in line with other reported analyses in a Medicare population [17]. While those in OPAL-HK initiated patiromer dosing as stratified by baseline K^+^ level, 92% of this real-world population started on the lowest dose of 8.4 g. The current study showed approximately half of patients on patiromer also had a RAASi prescription, which may partially be attributed to gaps in claims data (ie, failure to capture cash-paid transactions, including those filled under low-cost generic programs). Our real-world data also show substantial usage of patiromer in patients with CKD stage 5 or ESRD, while heart failure diagnoses were less frequent (20%) than in OPAL-HK (42%). Our study provides unique insight into prescribing patterns that otherwise may get generalized when compared historically to overlapping geography involved in clinical trials for the same condition.

Although this study assessed only prespecified comorbidities (CKD/ESRD, HF), real-world patients in this analysis were taking an average of nearly 10 daily-use medications before starting patiromer, which may serve as a reasonable surrogate of complex health status. Taking more than four or five daily-use medications is often considered polypharmacy when assessing medication-related risks (eg, harmful effects, drug interactions, hospitalizations) [18–20]. One-third of chronically ill patients struggle to afford food, medication, or both [21]. Those with comorbidities are likely to have added financial stress, which accounts for medication nonadherence in as much as 40% of patients with diabetes [21]. These financial obstacles may require essential provider time and development of a well-established, trusting relationship.

Our study has limitations. A large observational database of claims-level de-identified data provides associative information. On the other hand, it may uniquely offer insight on payer-agnostic prescription patterns and correlates with real-life socioeconomic indicators over large geographic/national areas. Similarly, the comorbid disease burden is derived from claims and can be specific but not very sensitive. Unlike registries, claims data may be incompletely captured or have gaps. Thus, this study cannot conclusively correlate adherence with patient outcomes. However, pharmacoepidemiologic analyses support broader correlations and highlight the differences between trial results and adoption of such into real-world practices, and they may inform interventional study planning to improve patient outcomes.

In summary, specific SDOH (unemployment, poverty, education, income) and health indicators (disability, comorbid CKD and HF) were associated with low PDC for patiromer use. Prescription abandonment was higher in patients prescribed higher initial patiromer doses, those with high out-of-pocket costs, those with disability, or those who were designated White. We successfully demonstrated that key SDOH play important roles in optimizing treatments for chronic illnesses. Considering SDOH barriers when designing interventions to improve drug access and adherence may be important to chronic hyperkalemia management and associated comorbidities. Furthermore, different approaches may be needed to overcome barriers to abandonment, those contributing to non-adherence, and other patient-specific factors. Further research on such individualized approaches is needed, as is further exploration on reasons patients abandon a first patiromer prescription or are non-adherent with chronic use. Lastly, examination of the impact of SDOH on prescription adherence may be extrapolated to other important drug classes that impact health outcomes, particularly emerging novel therapies in CKD.
